# Review of a Few Selected Theories of Plates in Bending

**DOI:** 10.1155/2014/291478

**Published:** 2014-10-28

**Authors:** Kaza Vijayakumar

**Affiliations:** Department of Aerospace Engineering, Indian Institute of Science, Bangalore 560012, India

## Abstract

The author's recent investigations on plate theories form the basis to review development of plate theories. In spite of several review articles on plate theories reported in the literature, the present work is essentially due to Jemielita's inspiring article (1993). It is shown that methods of analysis based on vertical displacement as domain variable deal with solution of associated torsion problem in bending of plates. It is essential to use vertical displacement as face variable instead of domain variable in proper analysis of bending problems.

## 1. Introduction

The author's recent investigations on plate theories form the basis to review development of plate theories. In spite of several review articles on plate theories reported in the literature, the present work is essentially due to Jemielita's inspiring article [1] “*On the winding paths of the theory of plates*.” Some noteworthy highlights of Jemielita's article are the progress in the formulation of theories of plates made in 1789–1988 has been carefully reviewed in a 217 page survey [2] encompassing more than 3000 items, about 1500 of them being discussed. An attempt was made to answer the general question,* “To study or to create*.*”* Facts in the development of plate theories have proved that one is supposed to study the previous works before creating a new theory. A significant observation in the referred treatise by Toudhunter and completed after his death by Pearson (1886) is that “the would-be researcher either wastes much time in learning the history of his subject, or else works away regardless of earlier investigations.” One could think that Pearson's words written on 23rd June 1886 became out of date in times of a stormy progress of communication but, unfortunately, it is not the case. For the past two hundred years a great number of theories of plates have been developed. Some of them fell flat, soon after their birth, others have still been applicable. For this period, pioneering papers have appeared indicating new directions of research, inspiring others to supplement and generalize the theories presented there. The old concepts are again recovered, the names of truly dedicated authors are forgotten, and their hypotheses and theories are often associated with other personalities, sometimes with scientists of a great authority.

A beginning towards the development of a plate theory by the present author was made years ago [3]. It was, in fact, due to the author's attention drawn (by his colleague, K. P. Rao) to Reissner's article [4]. At that time, his intention was confined only to the derivation of Reissner's expected sixth order equation of the form *D*∇^2^∇^2^ *w* − *C*∇^2^∇^2^∇^2^ *w* = *q*. He considered it superfluous to dwell in detail about other aspects mentioned in Reissner's article due to several investigators including scientists of great authority involved in the development of plate theories. The present paper is due to significant academic work resumed after a gap of more than 12 years since retirement in the year 1995.

*Scope of the Present Review*. Review of plate theories in this paper is explicitly limited to plates under bending* within small deformation theory of elasticity*. Any new theory, particularly, on bending of plates has to be with reference to elimination of deficiencies in the classical Kirchhoff's theory (well-known and widely used even today) as applied to the simple bending problem of simply supported (hard type) square plate. Analysis of plates with different geometries and material properties under different kinematic and loading conditions does not provide much scope for development of new theories other than those with the analysis of primary problems of a square plate. As mentioned by Ghugal and Shimpi [5] in their review paper, the development of refined structural theories for laminated plates (made up from advanced fiber reinforced composite materials) has their origins in the refined theories of isotropic plates.

## 2. Preliminaries of 3D Plate Problems of Isotropic Square Plate

Consider a square plate bounded within 0 ≤ *X*, *Y* ≤ *a*, and *Z* = ±*h* planes with reference to Cartesian coordinate system (*X*, *Y*, *Z*). Material of the plate is homogeneous and isotropic with elastic constants *E* (Young's modulus), *ν* (Poisson's ratio), and *G* (shear modulus) that are related to one other by *E* = 2(1 + *ν*)*G*. For convenience, coordinates *X*, *Y*, and *Z* and displacements (*U*, *V*, *W*) in nondimensional form *x* = *X*/*a*, *y* = *Y*/*a*, *z* = *Z*/*h*, (*u*, *v*, *w*) = (*U*, *V*, *W*)/*h*, and half-thickness ratio *α* = (*h*/*a*) are used. With the above notation and ⇄ indicating interchange, equilibrium equations in terms of stress components are (with 3D stress components as functions of *x*, *y*, and *z*)

(1) $$\begin{array}{c} \alpha \left(\sigma_{x,x}+\tau_{xy,y}\right)+\tau_{xz,z}=0\;⇄\left(x,y\right), \end{array}$$

(2) $$\begin{array}{c} \alpha \left(\tau_{xz,x}+\tau_{yz,y}\right)+\sigma_{z,z}=0 \end{array}$$

in which suffix after “,” denotes partial derivative operator.

In displacement based models, stress components are expressed in terms of displacements, via six strain-stress constitutive relations and six strain-displacement relations. These relations within the classical small deformation theory of elasticity are as follows:

*constitutive relations*:

(3) $$\begin{array}{c} E\varepsilon_{x}=\sigma_{x}-\nu \left(\sigma_{y}+\sigma_{z}\right)\;⇄\left(x,y\right), \end{array}$$

(4) $$\begin{array}{c} E\varepsilon_{z}=\sigma_{z}-\nu \left(\sigma_{x}+\sigma_{y}\right), \end{array}$$

(5) $$\begin{array}{c} \left[\tau_{xy},\tau_{xz},\tau_{yz}\right]=G\left[\gamma_{xy},\gamma_{xz},\gamma_{yz}\right]; \end{array}$$

*strain-displacement relations*:

(6) $$\begin{array}{c} \left[\varepsilon_{x},\varepsilon_{y},\varepsilon_{z}\right]=\left[\alpha u_{,x},\alpha v_{,y},w_{,z}\right],\\ \left[\gamma_{xy},\gamma_{xz},\gamma_{yz}\right]=\left[\alpha \left(u_{,y}+v_{,x}\right),u_{,z}+\alpha w_{,x},v_{,z}+\alpha w_{,y}\right]. \end{array}$$

For purposes of illustration, a simply supported square plate subjected to asymmetric load *σ*_*z*_ = ±(*q*_0_/2)sin(*πx*)sin(*πy*) and zero shear stresses along *z* = ±1 faces is considered throughout the present work. Conditions along *x* (and *y*) constant edges are

(7) $$\begin{array}{c} \sigma_{x}=0\;⇄\left(x;y\right);\;\;v=0\;⇄\left(v;u\right);\\ w=0. \end{array}$$

Nature of solutions from different methods of analysis is examined in the author's article (submitted elsewhere) with reference to the exact solution of the above mentioned text book problem. Methods of analysis are with reference to (i) exact solution of 3D problem in terms of displacements, (ii) sequence of 2D problems based on plate element equilibrium equations, and (iii) sequence of 2D problems based on infinitesimal element equilibrium equations.

## 3. Exact Solution with *w*(*x*, *y*, *z*) as Domain Variable

Equilibrium equations (1) and (2) in terms of displacements [6] are

(8) (1−2ν)(α2Δu+u,zz)+α(αu,xx+αv,yx+w,zx)=0mmmmmmmmmmmmmmmmmi⇄(x,y),(u,v),(1−2ν)(α2Δw+w,zz)+(αu,xz+αv,yz+w,zz)=0

in which Δ = (∂^2^/∂*x* ^2^ + ∂^2^/∂*y* ^2^) is plane Laplace operator.

From satisfying the above equations with displacements

(9) u=(A1sinh⁡⁡βz+A2zcosh⁡⁡βz)×cos⁡⁡πxsin⁡πy ⇄(x,y),(u,v),

(10) $$\begin{array}{c} w=\left(C_{1}\cosh\beta z+C_{2}z\sinh\beta z\right)\sin\pi x\sin\pi y \end{array}$$

in which $\beta =\sqrt{2}\alpha \pi$, one gets $C_{2}=\sqrt{2}A_{2}=(2\alpha \pi A_{1}-\beta C_{1})/(3-4\nu )$.

Zero shear stresses and vertical load condition along faces give

(11) C1=βtanh⁡β+2(1−ν)βtanh⁡β−(1−2ν)2A1=(1+ν)βsinh⁡⁡β+2(1−ν)cosh⁡⁡ββ(sinh⁡⁡βcosh⁡⁡β−β)(q02E).

Note that *α*(*v*_,*x*_ − *u*_,*y*_) denoted by *ω*_*z*_ is equal to zero from (9). As such, equilibrium equations (1) and (2) in terms of (*u*, *v*, *σ*_*z*_) with *E*′ = *E*/(1 − *ν* ^2^) and *μ* = *ν*/(1 − *ν*) become

(12) $$\begin{array}{c} E^{\prime }\alpha^{2}\Delta u+\mu \alpha \sigma_{z,x}+\tau_{xz,z}=0\;⇄\left(x,y\right), \end{array}$$

(13) $$\begin{array}{c} \alpha \left(\tau_{xz,x}+\tau_{yz,y}\right)+\sigma_{z,z}=0. \end{array}$$

It is found that the use of displacements (9) and (10) in solving the above equations gives the same solutions (11). With *α* = 1/6 and Poisson's ratio *ν* = 0.3 (for which solutions are available from various theories [7]), vertical displacement parameters in the illustrative example are

(14) $$\begin{array}{c} \left(\frac{E}{2q_{0}}\right)w\left(\frac{1}{2},\frac{1}{2},0\right)=3.49, \end{array}$$

(15) $$\begin{array}{c} \left(\frac{E}{2q_{0}}\right)w\left(\frac{1}{2},\frac{1}{2},1\right)=4.12 \end{array}$$

(earlier reported values [6] are not correct due to error in using *β*, unwittingly, in degrees instead of radians in evaluating hyperbolic terms).

Obviously, the above estimates correspond to those from the associate torsion problem since face deflection *w*_0*f*_ is greater than neutral plane deflection *w*_0*n*_. Note that neutral plane deflection is lower than that of face deflection by about 15% which decreases with decreasing thickness ratio.

## 4. Exact Solution with *w*_0_(*x*, *y*) as Face Variable

Exact solution of the present illustrative example was obtained earlier [8] with assumed in-plane displacements (9) and shear stresses, in place of *w*(*x*, *y*, *z*) in (10), in the form

(16) τxz=(C1cosh⁡⁡βz+C2zsinh⁡⁡βz)×cos⁡⁡πxsin⁡πy ⇄(x,y).

Normal stress *σ*_*z*_ from (13) is used in (12). Due to zero face shear stresses, face deflection *w*_0_(*x*, *y*) is evaluated by replacing [*u*_,*z*_, *v*_,*z*_] with [*u*,*v*]_*z*=1_ in shear stress-strain relations so that

(17) $$\begin{array}{c} \alpha w_{0}\left(x,y\right)=-\int {\left[udx+vdy\right]}_{z=1}. \end{array}$$

Vertical displacement parameters thus obtained are

(18) $$\begin{array}{c} \left(\frac{E}{2q_{0}}\right)w\left(\frac{1}{2},\frac{1}{2},0\right)=4.49;\\ \left(\frac{E}{2q_{0}}\right)w\left(\frac{1}{2},\frac{1}{2},1\right)=4.17. \end{array}$$

It is to be noted that the corresponding face deflection in the torsion problem is lower by about 1.2%. This difference in face deflections decreases with decrease in *α* and tends to zero in principle with *α* → 0. But it is constrained due to validity of small deformation theory restricted to lower bound of *α* from Kirchhoff's theory.

## 5. Displacements with Assumptions in the Kirchhoff's Theory [9]

We note that [*w*_0_, *u*_1_, *v*_1_] are primary variables in bending problems (shear stresses are also primary variables in the associated torsion problems). Correspondingly, transverse shear strains from strain-displacement relations are [*u*_1_ + *αw*_0,*x*_, *v*_1_ + *αw*_0,*y*_] with [*u*_1_, *v*_1_] that are negative of *α*[*w*_0,*x*_, *w*_0,*y*_] from assumption of zero transverse strains. Normal strain *ϵ*_*z*_ from constitutive relation with *e* = (*ϵ*_*x*_ + *ϵ*_*y*_) is proportional to *e*_1_  (*e* = *ze*_1_) which is not zero in the plate. It is neglected for design purposes in practical engineering problems. Its inclusion is, however, necessary for obtaining proper solutions of 3D problems.

Primary variables are determined from integrated equilibrium equations, that is, plate element equilibrium equations with reduced stiffness coefficient 2*E*/3(1 − *ν* ^2^). The term [*E*/(1 − *ν* ^2^)] is from semi-inverted in-plane strain-stress relations by neglecting *ϵ*_*z*_ in constitutive relations. In view of these assumptions, strain energy density is only due to in-plane stresses and strains. It can be easily shown that in-plane distributions of displacements thus obtained remain the same with displacements *z* ^2*k*^ *w*_2*k*_ and (*z* ^2*k*+1^/2*k* + 1)[*u*,*v*]_2*k*+1_  (*k* ≥ 1) with reference to the bending of plate subjected to the same kinematic and stress resultant conditions in the Kirchhoff's theory. Hence, *z*-distributions of displacements are not unique due to assumptions in Kirchhoff's theory. One should note, however, that vertical face deflection is the same for all *k* but [*u*, *v*] along faces are maximum from Kirchhoff's theory. Due to a priory satisfaction of zero face shear conditions, [*u*_1_, *v*_1_] are negative of *α*[*w*_0,*x*_, *w*_0,*y*_] resulting in a fourth order equation governing *w*_0_ along with two edge conditions instead of three edge conditions required in a 3D problem. Prescribed *τ*_*xy*_ along an edge is in the form of its tangential gradient contributing an artificial additional vertical shear. This additional shear is due to partial nullification of interior *τ*_*xy*_ in bending problem [10, 11].

### 5.1. Classical Sixth Order Theories

Minimum modification of Kirchhoff's theory has to be a sixth order theory rectifying lacuna in the theory along with facility of providing maximum face distribution of [*u*_1_, *v*_1_]. Reissner's pioneering work [12] discussed in detail later in the present paper was an attempt in this direction but not fully successful.

In Kirchhoff's theory, [*τ*_*xz*0_, *τ*_*yz*0_] are assumed to be zero due to zero face shear conditions. Normal stress *σ*_*z*_, though identically zero from equilibrium equation (2), is assumed to satisfy load condition *σ*_*z*_ = ±*q*/2 along the top and bottom faces of the plate. It is based on *f*_3_(*z*) = (1/2)(*z* − *z* ^3^/3) distribution a priory in Reissner's theory and from statically equivalent (reactive) stress in Kirchhoff's theory. In either case, transverse shear stresses correspond to parabolic *f*_2_(*z*) = (1/2)(1 − *z* ^2^) distributions. These distributions are also used through shear correction factor *k* ^2^ ( = 5/6) in FSDT based on Hencky's theory [13]. FSDT is a displacement based theory in which in-plane distributions [*τ*_*xz*0_, *τ*_*yz*0_] are used through constitutive and strain-displacement relations, whereas they are independent variables in Reissner's theory in applying Castigliano's theorem of least work. There is no need to use shear correction factor in Reissner's theory and its use is avoided in the displacement based Reddy's theory [14]. In stress based Reissner's theory, complementary strain energy due to *σ*_*z*_ involves cubic *f*_3_(*z*) distribution of *ε*_*z*_, whereas work done by the applied vertical load is associated with weighted vertical displacement. It must be noted here that Reddy's shear deformation theory is equivalent through change of variables to an earlier Reissner's displacement based theory [15] without the strain energy due to *σ*_*z*_ and both of them are equivalent to Ambartsumyan theory [16] proposed much earlier. As such, *σ*_*z*_ = (*zσ*_*z*1_) satisfying face load condition is ignored, whereas it plays an important role in rectifying lacuna in shear deformation theories as discussed later. In fact, these theories (which are based on plate element equilibrium equations with *w*_0_(*x*, *y*) as domain variable) correspond to approximate associated torsion problems.

In the illustrative example with *α* = 1/6 and *ν* = 0.3, FSDT gives (*E*/2*q*_0_)*w*_0_(1/2,1/2) = 3.69 and the corresponding value without shear correction factor is 3.41 [10]. The value 3.69 is much higher than the exact value 3.49 in (14). In the earlier mentioned submitted article, it is shown to be artificial from the following consideration.

In FSDT, the components (*τ*_*xz*2_, *τ*_*yz*2_) of reactive shear stresses are expressed earlier [10] in terms of (*τ*_*xz*0_, *τ*_*yz*0_) through distribution correction factor *β*_0_ = (5/2) obtained from

(19) $$\begin{array}{c} \left[\frac{1}{2}\left(1-z^{2}\right)\tau^{*}-\beta_{0}\tau \right]\left(1-z^{2}\right)dz=0, \end{array}$$

integrated from bottom to top faces of the plate giving *τ* ^*^ = *β*_0_ *τ* so that

(20) $$\begin{array}{c} \left(\tau_{xz}^{*},\tau_{yz}^{*}\right)=\left(\frac{5}{4}\right)\left(1-z^{2}\right)G\left(u_{1}+\alpha w_{0,x},v_{1}+\alpha w_{0,y}\right), \end{array}$$

resulting in shear energy correction factor *k* ^2^ = 5/6.

Adapting the above concept of deriving shear correction factor, higher order transverse shear terms with *f*_2*k*+2_(*z*) may be expressed in terms of preceding shear terms with *f*_2*k*_(*z*) through distribution correction factors *β*_2*k*_ so that

(21) $$\begin{array}{c} \tau_{xz}=\sum \left(1+\beta_{2k}\right)f_{2k}\tau_{xz2k}\;\left(k=0,1,2,3,\ldots \right)\;⇄\left(x,y\right). \end{array}$$

In such a case, solutions of plate element equations give shear strains [*u*_1_ + *αw*_0,*x*_, *v*_1_ + *αw*_0,*y*_] tending to [0,0] in the limit *k* → *∞*. Obviously, additional shear energy due to *β*_2*k*_ does not belong to the physical problem.

## 6. Review of Recent Theories

Displacement based Ambartsumyan's theory (equivalent Reissner's and Reddy's theories) consists of two-term representation of displacements. Only Kirchhoff's theory and FSDT are with one term representation of displacements (based on one variable *w*_0_(*x*, *y*) in the former and three primary variables [*w*_0_, *u*_1_, *v*_1_] in the latter). The present review is with reference to several shear deformation and higher order theories of this kind. Though most of them are used in the analyses of laminated and functionally graded plates, discussion of these theories is limited to the analyses of homogeneous plates. Further, we recall Jemielita's attempt in his inspiring paper to answer the general question, “To study or to create.” As such, discussion is restricted to the analyses of primary problems of a square plate since variations in geometry of the plate, material properties, presence of body forces, kinematic, and static boundary conditions belong to the category of study of various problems and do not play much role in creation of new plate theories.

### 6.1. Higher Order Theories

Thickness-wise distribution functions *f*_*n*_(*z*) generated from recurrence relations with *f*_0_ = 1, *f*_2*n*+1,*z*_ = *f*_2*n*_, and *f*_2*n*+2,*z*_ = −*f*_2*n*+1_ such that *f*_2*n*+2_(±1) = 0 are used in the recent investigations by the present author. They are (up to *n* = 5)

(22) $$\begin{array}{c} \left[f_{1},f_{2},f_{3}\right]=\left[z,\frac{1}{2}\left(1-z^{2}\right),\frac{1}{2}\left(z-\frac{z^{3}}{3}\right)\right],\\ \left[f_{4},f_{5}\right]=\left[\frac{\left(5-6z^{2}+z^{4}\right)}{24},\frac{z\left(25-10z^{2}+z^{4}\right)}{120}\right]. \end{array}$$

A 3D variable *F*(*x*, *y*, *z*) is expressed in the series form *F* = *f*_*n*_ *F*_*n*_(*x*, *y*) with sum *n* = 0,1, 2,3…. Displacements, strains, and stresses are expressed in the series form in four convenient groups:

(23) $$\begin{array}{c} \left[w,u,v\right]=f_{n}{\left[w,u,v\right]}_{n},\\ \left[\varepsilon_{x},\varepsilon_{y},\gamma_{xy},\varepsilon_{z}\right]=f_{n}{\left[\varepsilon_{x},\varepsilon_{y},\gamma_{xy},\varepsilon_{z}\right]}_{n},\\ \left[\sigma_{x},\sigma_{y},\tau_{xy},\sigma_{z}\right]=f_{n}{\left[\sigma_{x},\sigma_{y},\tau_{xy},\sigma_{z}\right]}_{n},\\ \left[\gamma_{xz},\gamma_{yz},\tau_{xz},\tau_{yz}\right]=f_{n}{\left[\gamma_{xz},\gamma_{yz},\tau_{xz},\tau_{yz}\right]}_{n}. \end{array}$$

Odd functions *f*_2*n*+1_(*z*) are replaced by

(24) $$\begin{array}{c} f_{2n+1}^{*}\left(z\right)=f_{2n+1}\left(z\right)-\beta_{2n-1}f_{2n-1}\left(z\right)\;\left(n\geq 1\right) \end{array}$$

in which *β*_2*n*−1_ = *f*_2*n*+1_(1)/*f*_2*n*−1_(1) so as to keep the associated 2D variables as free variables.

The above *f*(*z*) functions are even in vertical displacement *w* and odd in [*u*, *v*] in bending and associated torsion problems.

As discussed in the earlier work [8], Batista [17] generated coordinate functions *f*_*k*_(*z*) by the method of successive approximation satisfying zero transverse shear stress conditions along faces of the plate. With zero vertical load condition, he used plate element equilibrium equation so that *σ*_*z*_ is identically zero (physically invalid) in the domain. In obtaining solution in the limit, he derived Cheng's shear equation [18] (same as Levy's shear equation [19]) governing associated torsion problem in bending with respect to rotation about *x*-axis or *y*-axis in the case of rectangular plates. St. Venant's torsion problem is often used to illustrate sixth order theories though exact solution in the case *q* ≠ 0 requires expansion of *z* in sine series. In place of plate element equilibrium equations in Batista's work, Levy's refined theory was based on satisfying point-wise equilibrium equations using the following displacement field including sinusoidal functions (with sum on *n* = 0,1, 2,…):

(25) $$\begin{array}{c} w=z^{2n}w_{2n};\\ u=z^{2n+1}u_{n}+\varphi_{n}\sin\left(2n+1\right)\frac{\pi }{2}z\;⇄\left(u,v\right),\left(\varphi ,\psi \right). \end{array}$$

Kienzler [20] used power series in *ζ*  ( = *αz*) and Gol'denveizer and Kolos [21] used power series in *γ*  ( = *Z* = *hz*) in the derivation of plate equations. By using the recurrence relations mentioned above, one can generate *f*_*k*_(*αz*) and *f*_*k*_(*hz*) with integration limits 1 and *h*, respectively, so that *f*_*k*_ functions in (3) become homogeneous polynomials of degree *k* in (*α*, *ζ*) and (*h*, *Z*). It implies that *α* ^*k*^ and *h* ^*k*^ are scale factors of *f*_*k*_(*z*). 2D variables in Kienzler and Gol'denveizer and Kolos are connected to the present variables through linear transformations by equating coefficients of powers of *z*. In fact, plate element equilibrium equations from using polynomials in *z* (e.g., power series, Taylor series, orthogonal polynomials, and functions described in the beginning of this section) in higher order theories other than one-term representation of displacements in Kirchhoff's theory and FSDT become equivalent to one other with appropriate change of 2D variables.

### 6.2. Theories with 2-Term Representation of Displacements

Several two-term representations of displacements are used primarily intended to avoid the use of shear correction factor in FSDT. These displacements are of the form

(26) $$\begin{array}{c} w=w_{0}\left(x,y\right)+f_{,z}\left(z\right)w_{2}\left(x,y\right);\\ \left[u,v\right]=z\left[u_{1},v_{1}\right]+f\left(z\right)\left[u_{3},v_{3}\right]. \end{array}$$

Various theories are due to the choice of *f*(*z*) and six 2D variables in the above equation. Lower order theories based on a priory satisfying zero face shear conditions are

(27) $$\begin{array}{c} w=w_{0}\left(x,y\right)+f_{,z}\left(z\right)w_{2}\left(x,y\right);\\ \left[u,v\right]=-z\alpha \left[w_{0,x},w_{0,y}\right]+f\left(z\right)\left[u_{3},v_{3}\right] \end{array}$$

with assumed *f*(*z*) in different forms (to the author's best of knowledge) listed below:

(28) (i)  f3(z),(ii)  2πsin⁡π2z,(iii)  sinh⁡⁡z2−z2cosh⁡⁡12,(iv)  zexp⁡⁡(−12z2),(v)  (7z−4z3+z5)8,(vi)  (tan−1⁡z−z),(vii)  tan−1⁡(sin⁡π2z),(viii)  z[sec(rz2)−sec ⁡(r/2)1+(r/2)tan ⁡(r/2)]

(the parameter *r* is the shape parameter and its value is obtained as 0.1 in the postprocessing step by employing the inverse method [22]).

Note that (27) and (26) contain terms in Kirchhoff's theory and FSDT, respectively. Static equation (2) is satisfied in Kirchhoff's theory with *σ*_*z*_ ≡ 0 whereas it is satisfied with *σ*_*z*_ = (*zq*/2) in FSDT but ignored even in the constitutive relations. Corresponding transverse stresses in FSDT do not, however, participate in the in-plane equilibrium equations (1). To satisfy these equations even in the Kirchhoff's theory, one requires successive integrations in *z*-direction. All functions in (28) are approximations to this integration process. Theories based on plate element equations do not, however, rectify the lacuna in Kirchhoff's theory and FSDT.

Equations governing 2D variables from stationary property of total potential with each of the above functions form a tenth order system. They reduce to an eighth order system in shear deformation theories. Other eighth order systems are with *w*_2_(*x*, *y*) along with assumption of [*u*_3_, *v*_3_] as gradients *α*[*w*_2,*x*_, *w*_2,*y*_]. Another eighth order system is by replacing *w*_0_(*x*, *y*) + *f*_,*z*_(*z*)*w*_2_(*x*, *y*) with *w*_*b*_(*x*, *y*) + *w*_*s*_(*x*, *y*) along with *u* = −*α*[*zw*_*b*_ + *z* ^3^/3*w*_*s*_]_,*x*_ and *v* = −*α*[*zw*_*b*_ + *z* ^3^/3*w*_*s*_]_,*y*_. But it is equivalent to the above mentioned eighth order shear deformation theory with *w*_0_(*x*, *y*) = [*w*_*b*_(*x*, *y*) + *w*_*s*_(*x*, *y*)].

Apart from FSDT, one could consider several sixth order theories using *f*(*z*) in (28) in conjunction with displacements

(29) $$\begin{array}{c} w=w_{0}\left(x,y\right);\\ \left[u,v\right]=-z\alpha \left[w_{0,x},w_{0,y}\right]+f\left(z\right)\alpha \left[\psi_{,x},\psi_{,y}\right]. \end{array}$$

One should note from Lewinski's article [7] that estimates to the vertical displacement parameter from various 12th order theories are more or less equal to the exact neutral plane deflection 3.49 given in (14).

In the case of higher order theories applied to the present illustrative example, solutions of the plate element equations without shear correction factors would give increasing sequence of estimates to neutral plane deflection from 3.41 to 3.49. Corner reactions and vertical stress resultant *V*_*n*_(*s*) in Kirchhoff's theory are due to approximation of torsion problem but not widely believed bending problem. Moreover, FSDT describes an artificial approximation of torsion problem. Face and neutral plane deflections and solutions from various theories in the case of thin plates cluster together at lower bound of thickness ratio from Kirchhoff's theory for validity of small deformation theory. There is no reason to discuss about merits and demerits of these theories.

## 7. Poisson's Theory

In the author's recent investigations, *w*_0_(*x*, *y*) is used as face variable. New theory dealing with parabolic distribution of reactive transverse shear stresses is designated as “Poisson's theory of plates in bending” and its extension in which assumed transverse shear stresses are independent of *z*-coordinate, like *w*_0_(*x*, *y*) in Kirchhoff's theory and shear deformation theories, is designated as “Extended Poisson's theory” [6, 23].

In Kirchhoff's theory, basic variable is *w*_0_(*x*, *y*) and [*u*, *v*] are from [*γ*_*xz*_, *γ*_*yz*_] ≡ 0 in the plate. In FSDT, *w*_0_ is associated with *z*[*u*_1_, *v*_1_] through [*γ*_*xz*_, *γ*_*yz*_]. In these theories and other shear deformation theories, *σ*_*z*_ is neglected in constitutive relations. In the present analysis as in Kirchhoff's theory and FSDT, *σ*_*z*_ is neglected initially in the semi-inverted constitutive relations

(30a) $$\begin{array}{c} \sigma_{x}=E^{\prime }\left(\varepsilon_{x}+\nu \varepsilon_{y}\right)+\mu \sigma_{z}\;⇄\left(x,y\right), \end{array}$$

(30b) $$\begin{array}{c} \varepsilon_{z}=-\mu e+\frac{\left(1-2\nu \mu \right)\sigma_{z}}{E} \end{array}$$

in which *e* = (*ε*_*x*_ + *ε*_*y*_), *E*′ = *E*/(1 − *ν* ^2^), and *μ* = *ν*/(1 − *ν*).

In-plane variables (*u*_1_, *v*_1_) uncoupled from *w*_0_ are basic variables as in the earlier investigations [6, 11, 24]. Due to the condition *ω*_*z*_ = 0 required to decouple bending and torsion problems, one obtains reactive transverse stresses from thickness-wise integration of equilibrium equations

(31) $$\begin{array}{c} \tau_{xz}=E^{\prime }f_{2}\left(z\right)\alpha e_{1,x}\;⇄\left(x,y\right),\\ \sigma_{z3}=-E^{\prime }f_{3}\left(z\right)\alpha^{2}\Delta e_{1}. \end{array}$$

Constitutive relation gives *ε*_*z*_ = −*μf*_1_ *e*_1_. From satisfying face load condition, one gets the equation governing *e*_1_ as

(32) $$\begin{array}{c} \left(\frac{2}{3}\right)E^{\prime }\alpha^{2}\Delta e_{1}+q=0. \end{array}$$

In Poisson's theory, *e*_1_ in the above equation is replaced by *ψ*_2_(*x*, *y*) and it is solved with edge condition *ψ*_2_ = 0 in the simply supported plate problem. As mentioned earlier, the function *ψ*_2_ is related to normal strain *ε*_*z*_ and *ψ*_2_ = 0 implies *ε*_*z*_ = 0.

Equations governing *u*_1_ and *v*_1_ are given by

(33) $$\begin{array}{c} \alpha \left(u_{1,x}+v_{1,y}\right)=\psi_{2},\;\;\alpha \left(u_{1,y}-v_{1,x}\right)=0 \end{array}$$

so that

(34) $$\begin{array}{c} \alpha^{2}\Delta u_{1}=\alpha \psi_{2,x},\;\;\alpha^{2}\Delta v_{1}=\alpha \psi_{2,y}. \end{array}$$

Equations (33) are homogeneous without *ψ*_2_ and the corresponding *u*_1_ and *v*_1_ are conjugate harmonic functions coupled through *x* (and *y*) constant edge conditions with prescribed *T*_*x*_ and *T*_*xy*_

(35a) $$\begin{array}{c} u_{1}=0\;\text{or}\;E^{\prime }u_{1,x}=T_{x}\left(y\right)\;⇄\left(x,y\right),\left(u,v\right), \end{array}$$

(35b) $$\begin{array}{c} v_{1}=0\;\text{or}\;2Gv_{1,x}=T_{xy}\left(y\right)\;⇄\left(x,y\right),\left(u,v\right). \end{array}$$

From zero face shear conditions, one obtains *w*_0_(*x*, *y*) in terms of known [*u*_1_, *v*_1_] in the form

(36) $$\begin{array}{c} \alpha w_{0}\left(x,y\right)=-\int \left[u_{1}dx+v_{1}dy\right]. \end{array}$$

Vertical deflection thus obtained corresponds to face deflection (note that zero face shear conditions do not participate in the 3D domain equations unlike in Kirchhoff's theory). Its zero value along the edge requires simply a support to prevent vertical deflection of intersection of the face with wall of the plate. Such a support also ensures zero deflection of neutral plane along its edge. It is because the face and neutral plane deflections (*w*_0*f*_, *w*_*on*_) are one and the same since *w*(*x*, *y*, *z*) with (*w*_2_ + *ε*_*z*1_) = 0 can be expressed as

(37) w(x,y,z)=w0f(x,y)+f2(z)w2=w0n(x,y)−z2εz12.

However, *f*_2_(*z*) of second order correction *w*_2_ to vertical deflection *w* is parabolic; thereby, applied or reactive transverse shear stress along an edge is parabolic.

Equations (32) and (34) form a sixth order system for determination of *ψ*_2_, *u*_1_, and *v*_1_. Relevant three-edge conditions correspond to those required by Poisson. Hence, the theory is designated as* Poisson's theory of plates in bending*, parallel to Kirchhoff's theory. It is significant to note that the condition *w*_0_ = 0 prescribed along segments of the edge has no effect on the displacements [*u*_1_, *v*_1_, *w*_0_] obtained from Poisson's theory. It is to be noted that Poisson's theory gives an additional value of 0.27 to *w*(*x*, *y*, 1) due to *ε*_*z*1_ to the earlier *w*_0*f*_ estimate from Kirchhoff's theory.

The above described Poisson's theory is based on point-wise satisfaction of (1) and (2). Higher order corrections to the solutions from this theory are obtained through an iterative procedure [23] with initial solutions from Poisson's theory. At the first stage of iteration, displacements *f*_3_[*u*_3_, *v*_3_] (thereby, *ε*_*z*3_) consistent with *f*_2_[*τ*_*xz*2_, *τ*_*yz*2_], and reactive transverse stresses (*τ*_*xz*4_, *τ*_*yz*4_, *σ*_*z*5_) are obtained with *σ*_*z*5_ as a free variable by replacing *f*_5_ with *f*_5_^*^. Displacements [*u*_3_, *v*_3_] are modified in the form

(38) $$\begin{array}{c} u_{3}^{*}=u_{3}+\gamma_{xz2}-\alpha {\left(w_{2}-\varepsilon_{z1}\right)}_{,x}\;⇄\left(x,y\right),\left(u,v\right). \end{array}$$

The displacements [*u*_3_, *v*_3_] are determined through satisfaction of both static and integrated equilibrium equations. Higher order correction to *w*_0_ uncoupled from torsion is 1.26 so that total correction to the value from Kirchhoff's theory is about 1.54 giving *w*_0*f*_( = *w*_0*n*_) = 3.81.

*Supplementary Problem*. Neutral plane deflection *w*_0*n*_ is corrected from the solution of a supplementary problem with assumed displacements in the form

(39) [u,v,w]=[sin⁡(πz2)us3,sin⁡(πz2)vs3,  (π2)cos⁡⁡(πz2)ws2].

They are added as corrections to [*u*_3_^*^, *v*_3_^*^] obtained from iterative method so that [*u*, *v*] in supplementary problem are given by

(40) $$\begin{array}{c} \left[u,v\right]=\sin\left(\frac{\pi z}{2}\right)\left[\left(u_{3}^{*}+u_{s3}\right),\left(v_{3}^{*}+v_{s3}\right)\right]. \end{array}$$

Here, total correction over face deflection is about 0.66 giving a value of 4.46 which is very close to the exact value 4.49. Hence, it is safe to conclude that second order corrections in the displacements and transverse stresses from the iterative method serve the purpose of assessing data from Kirchhoff's theory and FSDT.

Solution for *w*_0_ consists of satisfying (i) zero face shear conditions and (ii) edge support conditions on *w*_0_. In Kirchhoff's theory, both these requirements are met through the single variable *w*_0_ governed by a fourth order equation. In Reissner's theory [15], average vertical displacement satisfies condition (ii) and, in FSDT, condition (i) is not satisfied, but its effect is included through shear energy correction factor. In any case, both of them are approximations to associated torsion problem [10]. The present analysis provides some clarity with regard to preliminary solution of primary flexure problem. It shows that determination of in-plane displacements, bending stresses, and reactive transverse stresses from integration of equilibrium equations is uncoupled from *w*_0_. However, edge condition *w*_0_ = 0 or contracted stress resultant *V*_*x*_ = *V*_*xo*_ in Kirchhoff's theory is different from the condition

(41) $$\begin{array}{c} \varepsilon_{z}=0\;\text{or}\;\tau_{xz2}=T_{xz}\;⇄\left(x,y\right) \end{array}$$

along constant *x* (and *y*) edge.

Error in the estimated value ( = 3.81) of *w*_0*f*_ is relatively high compared to the accuracy achieved in neutral plane deflection. In the earlier work [6], estimation of *w*_0*f*_ is further improved by modifying in-plane displacements (*u*_3_^*^, *v*_3_^*^) such that (*τ*_*xz*2_, *τ*_*yz*2_) are independent of *ε*_*z*1_. In the present example, correction to the face deflection changes to 1.43 so that face deflection value is 3.97 (= 2.27 + 0.27 + 1.43) which is under 4.7% from the exact value. The correction 1.43 to the face deflection is due to *ε*_*z*3_ from the constitutive relation. Determination of *ε*_*z*3_ in terms of (*σ*_*z*3_, *u*_3_, *v*_3_) involves lengthy algebra and arithmetical work.

The above analysis consists of a basic sixth order system and a supplementary fourth order system. Since the second order corrections are mainly due to inclusion of *σ*_*z*1_ in the in-plane constitutive relations, it is shown much simpler to find its effect from the extended Poisson's theory without consideration of higher order displacement components (*u*_3_, *v*_3_).

### 7.1. Extended Poisson's Theory

Assumptions in Kirchhoff's theory are eliminated in the extended Poisson's theory [6, 23]. Here, primary variables are [*u*_1_, *v*_1_, *ψ*_0_] in which *ψ*_0_(*x*, *y*) is solution of an auxiliary problem governing transverse stresses. These transverse stresses are independent of material constants so that they are global solutions and shown to be extremely useful in the analysis of homogeneous and laminated plates with anisotropic material constants in each ply [23].

In the auxiliary problem, transverse shears along faces of the plate are assumed in the form [*τ*_*xz*0_, *τ*_*yz*0_] = [*T*_*xz*_(*x*, *y*), *T*_*yz*_(*x*, *y*)]. It is more reasonable and practical to assume that they are the same in face parallel planes instead of *w*_0_(*x*, *y*). Correspondingly, [*τ*_*xz*0_, *τ*_*yz*0_] along constant *x* (and *y*) edges are

(42) $$\begin{array}{c} \tau_{xz0}=T_{xz}\left(y\right)\;⇄\left(x,y\right). \end{array}$$

They are more realistic edge conditions and more practical if they are independent of in-plane coordinates. In view of (2), [*τ*_*xz*0_, *τ*_*yz*0_] are expressed as

(43) $$\begin{array}{c} \left[\tau_{xz0},\tau_{yz0}\right]=\alpha \left[\psi_{0,x},\psi_{0,y}\right] \end{array}$$

so that *ψ*_0_(*x*, *y*) with *σ*_*z*_ = *zq*/2 is governed by

(44) $$\begin{array}{c} \alpha^{2}\Delta \psi_{0}+\frac{q}{2}=0. \end{array}$$

The above equation is to be solved with edge condition either *ψ*_0_ = 0 or its normal gradient equal to applied transverse shear stress along the edge. Assumed face stresses [*T*_*xz*_, *T*_*yz*_] correspond to [*τ*_*xz*0_, *τ*_*yz*0_] in (43) consistent with applied or reactive vertical shears along the edges. Here also, the function *ψ*_0_ is related to normal strain *ε*_*z*_ and *ψ*_0_ = 0 implies *ε*_*z*_ = 0.

With the aid of the above global solutions for transverse stresses, primary bending problem governing in-plane displacements is in terms of (12) and (13) along with statically equivalent transverse stresses and edge conditions

(45) $$\begin{array}{c} \left[\tau_{xz},\tau_{yz}\right]=f_{2}\left(z\right)\left[\tau_{xz2},\tau_{yz2}\right],\\ \sigma_{z}=\frac{zq}{2}+f_{3}\left(z\right)\sigma_{z3}. \end{array}$$

Here also, [*u*_1_, *v*_1_] are determined through satisfaction of both static and integrated equilibrium equations with modified displacements

(46) $$\begin{array}{c} u_{1}^{*}=u_{1}+\gamma_{xz0}-\alpha w_{0,x}\;⇄\left(x,y\right),\left(u,v\right) \end{array}$$

along with edge conditions

(47a) $$\begin{array}{c} u_{1}\left(y\right)=0\;\text{or}\;\;\sigma_{x1}\left(y\right)=T_{x1}\left(y\right)\;⇄\left(x,y\right),\left(u,v\right), \end{array}$$

(47b) $$\begin{array}{c} v_{1}\left(y\right)=0\;\text{or}\;\;\tau_{xy1}\left(y\right)=T_{xy1}\left(y\right)\;⇄\left(x,y\right),\left(u,v\right), \end{array}$$

(48) $$\begin{array}{c} \psi_{2}\left(y\right)=0\;\text{or}\;\;\tau_{xz2}\left(y\right)=\tau_{xz0}\left(y\right)\;⇄\left(x,y\right). \end{array}$$

If *q* = 0 and in-plane edge conditions are homogeneous, edge conditions (48) become the conditions used in obtaining global solution.

In the case of the present illustrative example, estimated face deflection (*E*/2*q*_0_) *w*_0*f*_ = 4.08 which is fairly close to the exact value 4.17. It shows that the solution for w from the present analysis provides proper correction to the estimation of face deflection. It is underestimated by 2.16% [11, 23].

*Supplementary Problem*. Corrective in-plane displacements in the supplementary problem are assumed in the form:

(49) $$\begin{array}{c} u_{s}=u_{1s}\sin\left(\frac{\pi z}{2}\right)\;⇄\left(u,v\right). \end{array}$$

In-plane distributions *u*_1*s*_ and *v*_1*s*_ are added as corrections to the known modified in-plane displacements (*u*_1_^*^, *v*_1_^*^), like in Poisson's theory, so that (*u*, *v*) in the supplementary problem are

(50) $$\begin{array}{c} u=\left(u_{1}^{*}+u_{1s}\right)\sin\left(\frac{\pi z}{2}\right)\;⇄\left(u,v\right). \end{array}$$

In the present illustrative example, estimated neutral plane deflection *w*_0*n*_ = 4.36(2*q*_0_/*E*) which is underestimated by 2.90% from the exact value 4.49.

Evaluation of neutral plane deflection involves *u*_1_ and *u*_1*s*_ associated with *f*_2_(*z*) and cos⁡(*πz*/2) distributions of transverse shear stresses, respectively. As such, the solution of the present auxiliary problem provides second order corrections to the solutions of primary problem. Moreover, wide variation of percentage variations of errors from 0.67 in *w*_0*f*_ to 4.80 in *w*_0*n*_ at the first stage of Iteration using (*u*_3_, *v*_3_) in Poisson's theory is reduced from 2.16 to 2.90 without using (*u*_3_, *v*_3_) in the extended Poisson's theory. One iteration using (*u*_3_, *v*_3_) satisfying both static and integrated equilibrium equations is expected to reduce this gap even further along with decreased percentage errors.* Significant implication of this observation is that the solution of auxiliary problem is necessary so as to obtain more or less uniform approximation to thickness-wise distributions of vertical displacement like in Kirchhoff's and shear deformation theories.*

## 8. Conclusions

(i) 3D equations in displacements and sequence of 2D problems with vertical displacement as domain variable correspond to associated torsion problems.
(ii) Kirchhoff's theory is a zeroth order shear deformation theory.
(iii) FSDT and higher order shear deformation theories with shear correction factors deal with artificial torsion problems.
(iv) It is mandatory to satisfy thickness-wise integrated equilibrium equations for determination of in-plane displacements. Facility of satisfying static equations improves solutions of these displacements.
(v) Poisson's theory and extended Poisson's theory are based on satisfaction of both static and integrated equilibrium equations.
(vi) Thickness-wise distribution of displacements in terms of polynomials *f*_*n*_(*z*) is not adequate for finding interior solutions of these displacements.
(vii) Solutions of auxiliary and supplementary problems are necessary to rectify lacuna in Kirchhoff's theory.
